# A review of studies on gut microbiota and levodopa metabolism

**DOI:** 10.3389/fneur.2023.1046910

**Published:** 2023-06-02

**Authors:** Zhe Zhong, Min Ye, Fuling Yan

**Affiliations:** ^1^Department of Neurology, Affiliated Zhongda Hospital, School of Medicine, Research Institution of Neuropsychiatry, Southeast University, Nanjing, China; ^2^Department of Neurology, Affiliated BenQ Hospital of Nanjing Medical University, Nanjing, Jiangsu, China

**Keywords:** Parkinson’s disease, levodopa, treatment, absorb, gut bacteria

## Abstract

Parkinson’s disease (PD) is the second most common neurodegenerative disease globally. Levodopa (L-dopa) has been the cornerstone for treating Parkinson’s since the 1960s. However, complications such as “wearing-off” and dyskinesia inevitably appear with disease progression. With the further development of microbiomics in recent years, It has been recognized that gut microbiota plays a crucial role in Parkinson’s disease pathogenesis. However, Little is known about the impact of gut microbiota in PD treatment, especially in levodopa metabolism. This review examines the possible mechanisms of gut microbiota, such as *Helicobacter pylori*, *Enterobacter faecalis*, and *Clostridium sporogenes,* affecting L-dopa absorption. Furthermore, we review the current status of gut microbiota intervention strategies, providing new insights into the treatment of PD.

## 1. Introduction

Parkinson’s disease (PD) is the most rapidly growing disorder worldwide and will affect 12 million people by 2024 (1). The pathology is characterized by dopaminergic neurodegeneration and misfolded synuclein accumulation. Since its introduction in the 1960s, L-dopa (L-dopa) has been the gold standard for treating Parkinson’s disease (2). Most Parkinson’s patients are eventually treated with L-dopa at different stages of the disease. Dopamine, the metabolite of L-dopa decarboxylase, is responsible for improving Parkinson’s disease symptoms. However, L-dopa is mainly decarboxylated in peripheral tissues, especially in the gastrointestinal tract; only 1%–5% of L-dopa eventually reaches the central system (3). On the one hand, peripheral L-dopa metabolism reduces its bioavailability and clinical effectiveness; on the other hand, it causes various complications such as orthostatic hypotension and arrhythmia (4). Therefore, L-dopa is routinely combined with decarboxylase inhibitors to reduce peripheral metabolism and enhance its bioavailability. However, even in the presence of decarboxylase inhibitors, up to half of levodopa is still metabolized in the periphery (5, 6). It is an urgent problem to increase the L-dopa bioavailability.

The gastrointestinal tract is an important site of mutual contact between humans and the environment. More than 100 trillion microorganisms reside in the gut, forming a complex community collectively known as the gut microbiota (7). These microorganisms have a wide range of physiological functions, host immunity regulation, and intestinal epithelial integrity maintenance (8). In particular, the microorganism contribution to drug metabolism is receiving increasing attention. Currently, There is already evidence that intestinal microorganisms such as Enterobacter faecalis, *Helicobacter pylori*, and *Clostridium sporogenes* can affect the metabolism and absorption of L-dopa (9–11). Moreover, interventions targeting gut microbiota have been proved to improve the clinical efficacy of L-dopaincluding antibiotics, dietary interventions, and Fecal microbiota transplantation (FMT) (12–14). Therefore, future researches are expected to conduct interventions for gut microbiota as the potential therapeutic target for PD. This review elaborates on the mechanisms of gut microbiota affecting L-dopa metabolism and emphasizes currently available intervention strategies targeting gut microbiota.

## 2. Methods

**Figure 1:**
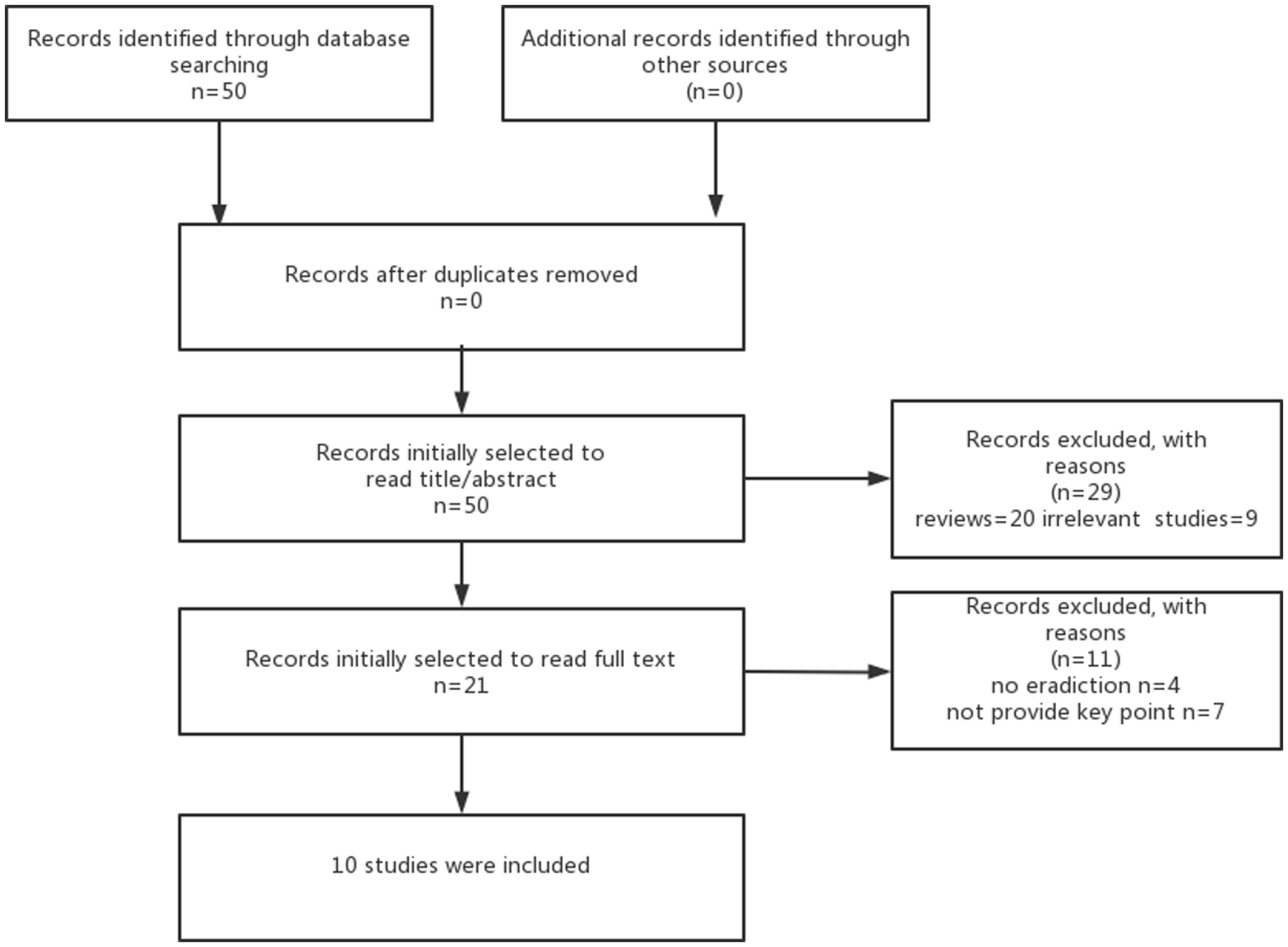
Flowchart of the study articles selected.

This review is aimed to discuss the link between gut microbiota and levodopa absorption and metabolism, focusing on the contribution of modulating gut microbiota to improve the efficacy of levodopa in clinical studies. Based on previous basic studies, we first identified the gut microbiota involved in levodopa metabolism (Table 1), and we then searched the articles between 2000 and 2021 based on the different combination of search terms “eradication bacteria” “levodopa” and “Parkinson’s disease”and obtained a total of 50 articles. Then, we excluded reviews and irrelevant studies after including a total of 21 studies after reading the abstract or title. Finally, a total of 10 studies were included based on the content of the studies after reading the full text. More details of the method are shown in Figure 1. The characteristics of 10 studies are shown in Table 2.

**Table 1:** Intestinal bacteria involved in the metabolism of levodopa.

Phylum	Family	Genus	Metabolism	References
Terrabacteria	Enterococcaceae	*Enterococcus faecalis*	Convert levodopa to dopamine by tyrosine decarboxylase	(15, 16)
Terrabacteria	Enterococcaceae	*Enterococcus faecium*	Convert levodopa to dopamine by tyrosine decarboxylases	(15, 16)
Terrabacteria	Eggerthellaceae	*Eggerthella lenta*	Convert dopamine to m-tyramine by molybdenum-dependent dehydroxylase	(15)
Proteobacteria	Helicobacteraceae	*Helicobacter pylori*	Delay gastric emptying time and change the PH	(17–19)
Terrabacteria	Eubacteriales	*Clostridium*	Deaminate the levodopa	(10)

**Table 2:** Characteristics of 10 studies.

Lnclude study	Year	Country	Sample size	Study design	Age	Male	Main findings	Limitations
Pierantozzi et al. (20)	2001	Italy	7	Non-randomized controlled studies	70.4 ± 6.3	NA	After HP eradication, mean concentration time increased (6584.7 ± 830.2 to 7871.0 ± 1169.5 ng/mL, *p* < 0.01), UPDRS III improved, (23.1 ± 8.8 vs. 33.9 ± 10.7; *p* < 0.01)	Small sample size; non-RCT study
Pierantozzi et al. (21)	2006	Italy	17	Randomized controlled trials	65.55 ± 7.6	16/34	After HP eradication, increase in levodopa uptake in PD patients along with improved UPDRSIII and prolonged “on-time”	Small sample size; allocation scheme was not described
Lee et al. (22)	2008	Korea	65	Prospective study	60.0 ± 9.5	36/65	After HP eradication, UPDRS-III scores slightly reduced (22.9 ± 7.4 to 22.3 ± 8.0 *p* > 0.05), “on-time” prolonged (210.0 ± 75.7 to 234.4 ± 66.5 min, *p* < 0.05)	Low accuracy of data and lack of credibility
Dobbs et al. (23).	2010	UK	30	Randomized controlled trials	63	19/30	After HP eradication, stride length improved (424 (379–468), *p* = 0.001)	No assessment of levodopa absorption;UPDRS was not performed； allocation scheme was not described
Dobbs et al. (24)	2013	UK	10	Prospective study	63	8/10	After HP eradication, stride increased (*p* = 0.001 each case). Flexor rigidity was unchanged	Small sample size; no assessment of levodopa absorption; non-RCT study
Fasano et al. (25)	2013	Italy	63	Prospective study	67.8 ± 8.5	31/63	After SIBO eradication, “off-time” and” on-time” improved (*p* = 0.04, *p* = 0.03); the levodopa absorption was not improved	The lactulose breath test (LBT) and glucose breath test (GBT) has lower specificity
Hashim et al. (26)	2014	Malaysia	76	Prospective study	65.1 ± 9.98	34/76	After HP eradication, “onset time” shortened by 14 min (*p* = 0.011). The “on-time” increased by 56 min at week 12 (*p* = 0.035). The UPDRSIII were significantly better (*p* = 0.0001)	Low accuracy of data and lack of credibility; short period of time (12w); single arm study design
Liu et al. (27)	2017	China	48	Prospective study	63.2 ± 7.44	9/22	After HP eradication,the UPDRS-III scores improved (25.9 ± 8.37 to 18.3 ± 8.38, *p* = 0.007)	No assessment of levodopa absorption; non-RCT study
Mridula et al. (28)	2017	India	36	Prospective studies	60	19/36	After HP eradication, UPDRS-III scores improved (22.3 to 15.3, *p* = 0.0001),“on-time”prolonged (2104.8 to 114.1, *p* = 0.005), “onset time” shortened (58.8 to 45.6, *p* = 0.007)	Serological tests not imply the presence of an active infection;unblinded open-label design
Tan et al. (13)	2020	Malaysia	67	Retrospective studies	66.0 ± 9.8	38/67	After HP eradication 12w; MDS-UPDRS scores not improved (mean difference 2.6, *p* = 0.089)	UPDRSIII was not performed； single-center study; no assessment of levodopa absorption

## 3. Gut microbiota and PD

Parkinson’s disease is multisystemic and imposes a severe burden on society. Apart from classic motor symptoms, non-motor symptoms are highly prevalent, including gastrointestinal and autonomic dysfunction (29, 30). Moreover, About 80% of PD patients experience gastrointestinal symptoms before experiencing motor symptoms (31). There is increasing evidence that gut microbiota can contribute to synucleinopathy and inflammation (32, 33). Therefore, Targeting the gut microbiota holds great promise for PD treatment.

### 3.1. Alterations of gut microbiota in PD

There have been more than 20 studies comparing the gut microbiota composition of PD patients with those of healthy controls. Moreover, more than 100 research have documented remarkable variations in α- and β-diversity as well as the relationship between disease severity and gut microbiota abundance (34, 35). Although there is no consensus on the microbiota composition in PD, It has been reported that Lactobacillus and Akkermansia have been enriched, while Prevotella and Faecalibacterium have been reduced in many studies (36–38). Notably, the microbiota composition of PD patients varies significantly due to their different regions and population. For example, the Lactobacillaceae family is universally enriched in western PD patients and less frequently found in china PD patients (39–41). However, whether gut microbiota alterations are a cause or a consequence of PD remains unclear.

### 3.2. Gut microbiota and PD pathogenesis

The pathogenesis of Parkinson’s disease appears to be strongly influenced by immunity. The inflammatory response in PD used to be thought to originate in the brain, however, there is a new concept that inflammation originates from the gastrointestinal tract. Since Braak (42) first proposed a gut-driven model, the gut microbiota facilitates the α-synuclein formation and transfer from the periphery to the brain. Increasing animal models confirmed that microbiota dysbiosis might trigger an inflammatory cascade response, leading to α-synuclein aggregation and dopaminergic neuron damage (43–45).

The gut microbiota contributes to immunity and inflammation through the following mechanisms: (i) gut microbiota dysbiosis affects microglia function. Gut microbiota dysbiosis can influence the microglia’s maturation and function, exacerbate neuroinflammation and promote the development of PD (46). (ii) Gut microbiota dysbiosis promotes abnormal activation of toll-like receptors (TLR). Lipopolysaccharides (LPS), endotoxins produced by gut microbiota, is an essential ligand of TLRs to increase microglia activation and release pro-inflammatory cytokines, causing an inflammatory response and aggravating substantia nigra (SN) damage (47, 48). (iii) Gut microbiota dysbiosis lead to the disturbance of metabolites. Short-chain fatty acids (SCFA), gut microbiota metabolites, contribute to energy metabolism and intestinal homeostasis. SCFA, especially butyrate, the primary energy substrate for colonic epithelial cells, is associated with intestinal barrier integrity (49, 50). Moreover, SCFA can promote microglia activation and α-syn aggregation in the central system (33).

## 4. Gut microbiota and L-dopa metabolism

### 4.1. Pharmacokinetics of L-dopa

L-dopa has been the cornerstone of PD drug treatment since the 1960s. After oral administration and passing through the stomach, L-dopa is absorbed in the duodenum and proximal jejunum. Notably, levodopa transport is mediated by an amino acid transport carrier, especially the SLC7A9-SLC3A1 (apical), SLC7A8-SLC3A2 (blood-brain barrier, basolateral), and SLC16A10 (basolateral) (51, 52).

Levodopa can compete with other neutral amino acids for binding carriers; therefore, avoid combining levodopa with dietary amino acids to reduce competition for transport in the intestine and blood-brain barrier. Most orally administered levodopa is metabolized in the periphery and distributed by skeletal muscles, only 1% of L-dopa can successfully enter the brain (53). Although combined L-dopa and decarboxylase inhibitors can reduce peripheral metabolism and increase effective levels in the brain, they still cannot meet clinical needs. Non-physiological administration will cause pulse-like stimulation of dopamine receptors on postsynaptic membranes, resulting in changes in striatal signal output pathways that ultimately lead to motor complications (54). Thus, improving the pharmacodynamics of L-dopa administration is essential for optimizing PD treatment.

### 4.2. Mechanisms of gut microbiota in L-dopa metabolism

#### 4.2.1. Enterococcus faecalis

Since the 1970s, There was already evidence that the gut microbiota can metabolize levodopa (55). Until recently, researchers identified the specific gut microbes, genes, and enzymes involved in levodopa metabolism. Tyrosine decarboxylase (TDC), mainly produced by *Enterococcus faecalis*, converts L-dopa to dopamine in the intestine (16) (15). Moreover, the microbiota-dependent conversion is not affected by aromatic L-amino acid decarboxylase (AADC) inhibitors. Furthermore, TDC expression was positively correlated with daily dose of levodopa and negatively correlated with plasma levodopa levels (16). Rekdal et al. (15) also confirmed that AADC inhibitors did not prevent microbiota-dependent conversion. In addition, they identified a particular compound, alpha-fluoromethyltyrosine (AFMT), which effectively blocks the microbiota-dependent conversion. These two seminal studies bring new insight into traditional AADC inhibitor supplementary therapy. We believe we can further reduce peripheral metabolism, increase central levodopa levels, and improve clinical efficacy by modulating gut microbiota in the future.

#### 4.2.2. Helicobacter pylori

It has been proven that *Helicobacter pylori* (HP) can affect L-dopa’s efficacy, and symptoms improved after HP eradication (20, 21, 56). Lee et al. (22) conducted an observational study of HP and PD. The study found a considerable increase in L-dopa “on-time” after HP eradication in PD patients (58.1 ± 25.6 ~ 234.4 ± 66.5 min). Similar results were reported in another study, the onset time of L-dopa was shortened by 14 min, and the average on-time was increased by 38 min after 12 weeks of HP eradication. Moreover, the unified Parkinson’s disease rating scale (UPDRS) and Parkinson’s disease questionnaire 39 (PDQ-39) were significantly improved (26). Liu and colleagues (27) conducted long-term research (one year) on HP eradication and reported similar results. Patients with HP-positive PD had significantly lower UPDRS scores one year after HP eradication, especially in the fine motor aspect of the hands and feet. We speculate that the mechanisms of HP affect levodopa efficacy in the following ways: one possibility is that HP infection may delay gastric emptying, resulting in delayed L-dopa entry and impaired L-dopa transport in the duodenum (17). In addition, HP can use L-dopa as a supplement for its own growth to maintain its ecological niche (57). Finally, The vitro studies have shown that HP adhesins can directly interact with L-dopa, leading to a reduction in the effective concentration (19, 58). However, in a recent double-blind controlled trial, there was no remarkable improvement in motor and non-motor symptoms after HP eradication (13). This result contradicts the previous study, and further larger samples with strict screening criteria and follow-up are needed to demonstrate the therapeutic importance of HP eradication in PD patients. Although it is questionable whether HP eradication provides long-term benefits for PD, we need to be aware that HP infection can cause a variety of gastric diseases and even gastric cancer. Therefore, it is advocated that PD patients with motor fluctuations or wearing-off should go for HP detection and treatment.

#### 4.2.3. Clostridium sporogenes

As previously mentioned, the vast majority is delivered to the circulatory system by intestinal amino acid transport carriers and only 10% of unabsorbed residual L-dopa remains in the intestine as a substrate for other bacterial species (3). However, researchers found that microbiota can interact with residual levodopa and influence intestinal homeostasis through its metabolites. van Kessel et al. (10) found that *Clostridium sporogenes* can deaminate L-dopa to 3-(3,4-dihydroxy phenyl) propionic acid that can inhibit ileal motility *in vitro*. It is crucial to identify the potential effects of bacteria on drug metabolism to improve efficacy and reduce unwanted side effects.

#### 4.2.4. Small intestinal bacterial overgrowth

Small intestinal bacterial overgrowth (SIBO) refers to gut dysbiosis marked by a high number or unusual type of bacteria in the small intestine. Although SIBO is not a disease, it can cause a wide range of gastrointestinal symptoms, for example, abdominal distension, burping, flatulence, diarrhea, and constipation. Eleven studies had reported the prevalence of SIBO in PD ranging from 46%–67% (59). SIBO has been reported to have more severe motor functions and longer off-time compared to PD without SIBO (25, 60). Furthermore, Significant improvement in motor dysfunction in patients with Parkinson’s disease after the SIBO eradication in a non-randomized trial (25). The same results were also reported in another prospective study, where motor function improved (*p* < 0.05)after the correction of SIBO with FMT (61). SIBO-induced peripheral inflammation increases intestinal permeability and bacterial translocation, triggering microglia activation and enhancing alpha-synuclein aggregation, which plays an important role in the pathogenesis of PD (62, 63). In addition, SIBO-induced inflammation can act on enterochromaffin-like cells and cause delayed gastric emptying (64). More importantly, SIBO can cause inflammation of the proximal jejunum (the main absorption site of levodopa) and impair levodopa absorption (65). In conclusion, the eradication of SIBO serves as a potential disease-modifying intervention to slow the progression of PD and optimize Parkinson’s treatment by improving L-dopa efficacy.

## 5. Potential strategies for targeting the gut microbiota

### 5.1. New formulations of L-dopa

Obviously, Traditional oral formulations of levodopa no longer meet current needs, and new formulations hold great promise for the future. Accordion Pill CD-L-dopa, a new oral formulation of gastric retention extended-release with unique multi-layer planar structure, retains in the stomach for up to 12 h and improves daily “off-time” (66). However, whether the drug is affected by delayed gastric emptying and gut microbiota is not known. Additionally, Non-oral formulations have been extensively studied. (i) intestinal route: L-dopa-carbidopa intestinal gel (LCIG), continuously transported in the jejunum *via* an external pump, can effectively reduce motor fluctuations and improves “on” time (67). (ii) Transcutaneous route: ND0612 is administered subcutaneously *via* a pump-patch device for steady plasma levels. It has been reported to be well tolerated and ease dyskinesia (68). (iii) Inhalation route: CVT-301 is a powder formulation of levodopa delivered by a breath-driven inhaler. CVT-301 rapidly increases the plasma concentration of L-dopa with less fluctuation in blood concentration (69). However, it is worth noting that whether intranasal microorganisms affect the efficacy of inhaled formulations deserves further study. Overall non-oral formulations allow levodopa to enter the body’s circulation more quickly and reliably than traditional oral formulations (new formulations of L-dopa in Table 3).

**Table 3:** L-dopa new formulations.

Drug	Trial status	Route	Mechanism of action	Adverse events	References
IPX066	Phase III FDA/EU approved	Oral	L-dopa/carbidopa-containing formulation	Nausea, vomiting, headache, dizziness, insomnia	(70)
AP L-dopa/CD	Phase III ongoing	Oral	retention of multilayer films containing immediate-release (IR) carbidopa (CD) and immediate and controlled-release L-dopa	n/a	(66)
L-dopa/carbidopa intestinal gel (LCIG)	Phase III FDA/EU approved	Intestinal route	Continuous intestinal delivery of L-dopa/carbidopa	Device-related complications are frequent	(67)
L-dopa/entacapone/carbidopa intestinal gel (LECIG)	Phase III FDA/EU approved	Intestinal route	Continuous intestinal delivery of L-dopa/carbidopa + COMT-I	Procedure and device-related complications are frequent	(71)
ND0612	Phase III ongoing	Transcutaneous route	Continuous subcutaneous L-dopa/carbidopa pump	Infusion site reactions	(68)
ABBV-951	Phase I	Transcutaneous route	Subcutaneous delivery of L-dopa/carbidopa phosphate prodrug	n/a	(72)
CVT-301	Phase III ongoing	Inhalation route	Inhaled L-dopa preparation	n/a	(69)

### 5.2. Supplement targeting the gut microbiota

Novel strategies targeting gut microbiota in treating PD focus on normalizing abnormal bacteria and reducing neuroinflammation and neuronal damage, slowing the course of the disease. Several studies have shown that dietary interventions, probiotics, and prebiotics are beneficial in treating PD (73–75). Dietary interventions and supplementing omega-3 fatty acids and vitamins can accelerate CNS barrier repair, reduce neuroinflammation, and slow down neurodegenerative processes. Evidence suggests that omega-3 fatty acids indirectly block the activation of TLR4 and other TLRs, which reduces mitochondrial dysfunction and the accumulation of synaptic nuclides (76). In addition, it was found that omega-3 fatty acids could attenuate the damage of dopaminergic neurons by LPS by inhibiting NF-κB activation (77). Furthermore, probiotics have been shown to increase glucose metabolism, reduce peripheral inflammation, and improve motor and non-motor function in pre- and post-clinical studies (78, 79). Besides, FMT can also rebuild gut microbiota and maintain intestinal homeostasis. In our previous study, FMT reduced the expression of α-syn and inhibited the activation of microglia in SN (80). Specific decarboxylase inhibitors AFMT, engineered gut bacteria, live bacteria drugs, and synthetic bacteria, may also have potential therapeutic value for PD. Consequently, these novel potential treatment approaches must undergo carefully designed randomized clinical trials to determine their benefits and safety.

### 5.3. Antibiotics targeting the gut microbiota

As mentioned above, It is promising to reduce drug metabolism and inflammation in the intestine by inhibiting specific bacteria. Antibiotics are chemicals that can inhibit or eliminate certain microorganisms. Moreover, antibiotics have now been repurposed because of their anti-inflammatory and neuroprotective properties. Rifaximin is a broad-spectrum antibiotic that is considered an effective treatment for SIBO due to its broad spectrum (81). In addition, Rifampicin has been found to cross the BBB in animal models, prevent neurodegeneration of nigrostriatal dopaminergic neurons, reduce microglial inflammation, and improve mitochondrial dysfunction (82, 83). Tetracyclines are broad-spectrum antibiotics and can eliminate microbiota in high concentrations. It could reduce pro-inflammatory molecules and inhibit matrix metalloproteinase activity (84, 85). Moreover, ceftriaxone (CEF) is a b-lactam antibiotic most frequently used in hospital-acquired infections. Recent studies have highlighted the therapeutic effects of CEF on neurodegenerative diseases. For example, CEF can modulate inflammation and gut microbiota and has neuroprotective effects in PD mice (86). However, It is unclear which antibiotic type and dose is appropriate for patients with PD.

## 6. Conclusion

L-dopa is the primary medication for PD and can improve motor symptoms in the different stages of the disease. However, as the disease progresses, there are complications such as decreased efficacy and dyskinesia, and L-dopa will not continue to delay progression. The interaction between gut bacteria and drugs may offer new insights into treating Parkinson’s disease.

Based on this perspective, microbiome research offers a bright future for the field. Looking toward the future, we should explore various study designs that manipulate the gut microbiota, including using selective antibiotics to reduce the abundance of *TDC*, using probiotics to repopulate gut bacteria, and exploring the potential role of fecal bacteria transplantation. There is limited evidence supporting these beneficial effects in preclinical and clinical settings. Thus, it requires more high-quality, multicenter clinical trials to determine efficacy and safety.

## Author contributions

ZZ contributed to the writing and conceptualization of the manuscript, including all figures and data involved. MY and FLY revised the content and format of the manuscript and made equally important contributions. All authors contributed to the article and approved the submitted version.

## Conflict of interest

The authors declare that the research was conducted in the absence of any commercial or financial relationships that could be construed as a potential conflict of interest.

## Publisher’s note

All claims expressed in this article are solely those of the authors and do not necessarily represent those of their affiliated organizations, or those of the publisher, the editors and the reviewers. Any product that may be evaluated in this article, or claim that may be made by its manufacturer, is not guaranteed or endorsed by the publisher.
